# The Heat Partition Ratio during Braking in a Functionally Graded Friction Couple

**DOI:** 10.3390/ma15134623

**Published:** 2022-06-30

**Authors:** Aleksander Yevtushenko, Katarzyna Topczewska, Przemysław Zamojski

**Affiliations:** Faculty of Mechanical Engineering, Bialystok University of Technology (BUT), 45C Wiejska Street, 15-351 Bialystok, Poland; a.yevtushenko@pb.edu.pl (A.Y.); p.zamojski@pb.edu.pl (P.Z.)

**Keywords:** heat partition ratio, functionally graded materials, frictional heating, temperature, braking

## Abstract

The theoretical scheme for determining the heat partition ratio (HPR) in a friction couple made of functionally graded materials (FGMs) was proposed. As a result, the formula for the calculation of the HPR was found, which depends on the thermal properties and the parameters of the material’s gradient. In specific cases of these parameters, the known formulas for estimating the HPR for homogeneous materials were obtained. Calculations were carried out for the friction couple consisting of the following two-component FGMs: Al_2_O_3_–Cu (first body) and ZrO_2_–Ti–6Al–4V (second body), under the conditions corresponding to a single braking with a constant deceleration. It was established that the vast majority (almost 90%) of heat that was generated by friction was absorbed by the first body in the selected couple. The possibilities of using the obtained results were discussed herein.

## 1. Introduction

Functionally graded materials (FGMs) are materials in which, along at least one specific direction, a continuous change in the functional or structural properties has been obtained in a selected technological process. The possibilities of using graded materials in technology seem to be almost unlimited. Proposals of possible FGM applications appear for many industries, such as the following: optical, energy [1,2], aviation [3,4], medical [5], etc. Moreover, they have been used in friction elements, including braking systems [6,7].

The significant influence of temperature on the friction and wear characteristics of friction materials is well known and has been studied by many researchers who are involved in determining the temperature mode of disc brakes [8,9,10]. The basis for establishing the temperature are the solutions (analytical, numerical, or analytical–numerical) to the thermal problems of friction, i.e., the boundary-value problems of heat conduction with two specific boundary conditions on the contact surface of the pad and the disc [11,12]. One of them, the energetic solution, determines the equality of the specific friction power q and the sum of the heat flux intensities $q_{l}$,l=1,2 that are directed along the normal surface to the contact surface towards the insides of the friction elements $q_{1}+q_{2}=q$. Whereas the second solution concerns the type of thermal contact—with (imperfect) or without (perfect) consideration of the thermal resistance on the friction surfaces. In the latter case, the surface temperatures of the friction elements are the same. The coupling of the temperature fields of both of the friction elements through the above-mentioned boundary conditions means that obtaining the analytical [13,14,15,16] or the numerical [17,18,19] solutions of the thermal problems of friction requires the performance of complex mathematical transformations or long-term calculations.

In order to reduce these difficulties, there is also another approach to estimate the temperature of the braking systems, on the basis of solutions to the thermal problems of friction. It is based on a virtual separation of the friction pair elements and the subsequent replacement of the frictional interaction on their working surfaces with the heat fluxes of the following intensities: $q_{l}=\alpha_{l}q$, l=1,2, $\alpha_{1}=\alpha$, and $\alpha_{2}=1-\alpha$, respectively, where α is the heat partition ratio [20,21]. Reviews of the experimental methods, as well as the theoretical methods, for determining the HPR in braking systems have been developed [22,23,24,25]. The theoretical methods primarily rely on the fact that the temperature field, which is first found by means of analytical solutions, contains *a priori* unknown coefficient α, which is then determined from the condition of the equality of the maximum or the mean temperature on the friction surfaces of the pad and the disc. As a result, the formulas for the calculation of α contain the thermo-physical properties of the friction materials and, in some cases, the operating parameters of the process. Substituting thus determines the value of α back into the analytical or the numerical solutions, and the temperature mode of the braking system is estimated.

It should be noted that the formulas that have been obtained so far for determining the HPR, based on the analytical solutions to the thermal problems of friction, concern homogeneous materials [26,27,28,29,30]. The proportion of the heat distribution by means of the HPR between polymer and steel elements was established experimentally [31]. The aim of this study is to obtain the theoretical formulas for estimating the HPR during the braking of friction pair elements that are made of functionally graded materials (FGMs). One of the proposed applications of the results could be the determination of the bulk temperature of friction elements that are made of thermally sensitive FGM, during a single braking process [32] or a repetitive short-term braking mode [33].

## 2. Heating of the FGM Semi-Space by the Heat Flux with Constant Intensity

**Figure 1:**
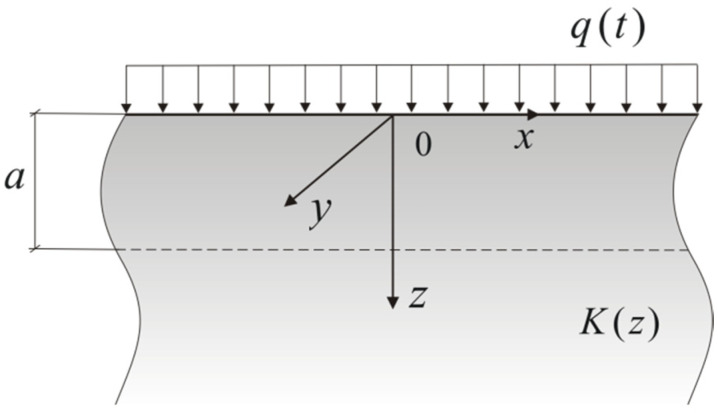
Scheme of the heating of the FGM semi-space.

Consider a temperature of T of a semi-limited body z≥0, which is uniform and equal to $T_{0}$ at the initial point in time of t=0. The body is made of a functionally graded material (FGM) with the thermal conductivity of K, increasing exponentially in the positive direction of the axis z as follows:

(1) $$K(z)=K_{0}e^{\gamma z},z\geq 0,K_{0}\equiv K(0),$$

where γ>0 is the gradient of the material. Next, the semi-space is heated on the outer surface as z=0 by the heat flux with a constant intensity of $q=q_{0}$ in time as t>0 (Figure 1).

The transient temperature field of the semi-space was searched in following the form:

(2) $$T(z,t)=T_{0}+\Theta (z,t),\;z\geq 0,\;t\geq 0,$$

where the temperature rise of Θ(z,t) was determined from the solution to the following boundary-value heat conduction problem:

(3) $$\frac{\partial }{\partial z}\left[K(z)\frac{\partial \Theta (z,t)}{\partial z}\right]=\rho c\frac{\partial \Theta (z,t)}{\partial t},\;z>0,\;t>0,$$

(4) $${K_{0}\frac{\partial \Theta (z,t)}{\partial z}|}_{z=0^{+}}=-q_{0},\;t>0,$$

(5) Θ(z,t)→0, z→∞, t>0,

(6) Θ(z,0)=0, z≥0,

and ρ, c are the density and the specific heat capacity of the material, respectively.

The following dimensionless variables and parameters were introduced:

(7) $$\zeta =\frac{z}{a},\;\gamma =\frac{\gamma^{*}}{a},\;\tau =\frac{kt}{a^{2}},\;k=\frac{K_{0}}{c\rho },\;q^{*}=\frac{q}{q_{0}},\;\Theta^{*}=\frac{\Theta }{\Theta_{0}},\;\Theta_{0}=\frac{q_{0}a}{K_{0}},$$

where a is the effective heating depth, i.e., the distance from the heated surface, at which the temperature is equal to 5% of the maximum temperature on the heated surface [2]. Taking into account the designations (7), the problem (3–6) was written in following the form:

(8) $$\frac{\partial^{2}\Theta^{*}(\zeta ,\tau )}{\partial \zeta^{2}}+\gamma^{*}\frac{\partial \Theta^{*}(\zeta ,\tau )}{\partial \zeta }-e^{-\gamma^{*}\zeta }\frac{\partial \Theta^{*}(\zeta ,\tau )}{\partial \tau }=0,\;\zeta >0,\;\tau >0,$$

(9) $${\frac{\partial \Theta^{*}(\zeta ,\tau )}{\partial \zeta }|}_{\zeta =0}=-1,\;\tau >0,$$

(10) $$\Theta^{*}(\zeta ,\tau )\to 0,\;\zeta \to \infty ,\;\tau >0,$$

(11) $$\Theta^{*}(\zeta ,0)=0,\;\zeta \geq 0.$$

Applying the following Laplace integral transform [34]:

(12) Θ¯∗(ζ,p)≡L[Θ∗(ζ,τ); p]=∫0∞Θ∗(ζ,τ)e−pτdτ, Rep≥0,

to the boundary-value problem (8)–(11), the following were obtained:

(13) $$\frac{d^{2}{\bar{\Theta }}^{*}(\zeta ,p)}{d\zeta^{2}}+\gamma^{*}\frac{d{\bar{\Theta }}^{*}(\zeta ,p)}{d\zeta }-pe^{-\gamma^{*}\zeta }{\bar{\Theta }}^{*}(\zeta ,p)=0,\;\zeta >0,$$

(14) $${\frac{d{\bar{\Theta }}^{*}(\zeta ,p)}{d\zeta }|}_{\zeta =0^{+}}=-\frac{1}{p},$$

(15) $${\bar{\Theta }}^{*}(\zeta ,p)\to 0,\;\zeta \to \infty .$$

The solution to the boundary problem (13)–(15) takes the following form:

(16) $${\bar{\Theta }}^{*}(\zeta ,p)=e^{-\frac{1}{2}\gamma_{1}^{*}\zeta }\;\frac{\varphi (\zeta ,p)}{\Phi (p)},\;\zeta \geq 0,$$

where

(17) $$\varphi (\zeta ,p)=\;I_{1}\left(\frac{2}{\gamma^{*}}\sqrt{p}\;e^{-\frac{1}{2}\gamma^{*}\zeta }\right),$$

(18) $$\Phi (p)=p\sqrt{p}\;I_{0}\left(\frac{2}{\gamma^{*}}\sqrt{p}\right),$$

$I_{k}(x)$, k=0,1 are the modified Bessel functions of the first kind [35]. Differentiating the function (18) with consideration of the relation of ${I^{\prime }}_{0}(x)=I_{1}(x)$, the following was found:

(19) $$\Phi^{\prime }(p)=\frac{3}{2}\sqrt{p}\;I_{0}\left(\frac{2}{\gamma^{*}}\sqrt{p}\right)+\frac{p}{\gamma^{*}}I_{1}\left(\frac{2}{\gamma^{*}}\sqrt{p}\right).$$

The transition from the space of the transforms to the originals in the solution (16)–(19) was carried out on the basis of the Vashchenko-Zakharchenko theorem [36,37] as follows:

(20) $$\Theta^{*}(\zeta ,\tau )=e^{-\frac{1}{2}\gamma^{*}\zeta }\left[\underset{p\to 0}{\lim}\frac{\varphi (\zeta ,p)p}{\Phi (p)}+\sum_{n=1}^{\infty }\frac{\varphi (\zeta ,p_{n})}{\Phi^{\prime }(p_{n})}e^{p_{n}\tau }\right],\;\zeta \geq 0,\;\tau \geq 0,$$

where

(21) $$I_{0}\left(\frac{2}{\gamma_{1}^{*}}\sqrt{p_{n}}\right)\equiv 0,\;n=1,2,\ldots$$

By using the expressions of the modified Bessel functions [35] as follows:

(22) $$I_{0}(x)≅1+\frac{x^{2}}{4}+\frac{x^{4}}{64}+\ldots ,\;I_{1}(x)≅\frac{x}{2}+\frac{x^{3}}{16}+\ldots$$

and by limiting them to the first components, the following representations of functions (17) and (18) for the small values of the parameter p were obtained:

(23) $$\varphi (\zeta ,p)≅\;\frac{1}{\gamma^{*}}e^{-\frac{1}{2}\gamma^{*}\zeta }\sqrt{p},\;\Phi (p)≅p\sqrt{p}.$$

Taking into account the Formulas (23) and relationships [35] as follows:

(24) $$I_{0}(x)=J_{0}(ix),\;I_{1}(x)=-iJ_{1}(ix),\;i\equiv \sqrt{-1},$$

where $J_{k}(x)$, k=0,1 are the Bessel functions of the first kind, solutions (20) and (21) were written in the following form:

(25) $$\Theta^{*}(\zeta ,\tau )=\frac{1}{\gamma^{*}}e^{-\frac{1}{2}\gamma^{*}\zeta }\left[e^{-\frac{1}{2}\gamma^{*}\zeta }-4\sum_{n=1}^{\infty }\frac{J_{1}(\mu_{n}e^{-\frac{1}{2}\gamma^{*}\zeta })}{\mu_{n}^{2}J_{1}\left(\mu_{n}\right)}e^{-\lambda_{n}\tau }\right],\;\zeta \geq 0,\;\tau \geq 0,$$

where

(26) $$\lambda_{n}={\left(\frac{1}{2}\gamma^{*}\mu_{n}\right)}^{2},$$

(27) $$J_{0}(\mu_{n})\equiv 0,\;n=1,2,\ldots$$

By adopting ζ=0 in the solution (25)–(27), the dimensionless temperature rise on the heated surface was found in the following form:

(28) $$\Theta^{*}(\tau )\equiv \Theta^{*}(0,\tau )=\frac{1}{\gamma^{*}}\left(1-4\sum_{n=1}^{\infty }\frac{e^{-\lambda_{n}\tau }}{\mu_{n}{}^{2}}\right),\;\tau \geq 0.$$

Verification of the developed model was carried out by checking the boundaries (9), (10), and the initial (11) conditions. For this, by differentiating the solution (25) with respect to the spatial variable ζ, and taking into account the relationship [35] as follows:

(29) $${J^{\prime }}_{1}(x)=J_{0}(x)-x^{-1}J_{1}(x)$$

the following was found:

(30) $$\frac{\partial \Theta^{*}(\zeta ,\tau )}{\partial \zeta }=-e^{-\gamma^{*}\zeta }\left[1-2\sum_{n=1}^{\infty }\frac{J_{0}(\mu_{n}e^{-\frac{1}{2}\gamma^{*}\zeta })}{\mu_{n}J_{1}\left(\mu_{n}\right)}e^{-\lambda_{n}\tau }\right],\;\zeta \geq 0,\;\tau \geq 0.$$

Approaching Formula (30) to the limit of ζ→0, with consideration of the fact that $\mu_{n}$ are the roots of Equation (27), the following was achieved:

(31) $${\frac{\partial \Theta^{*}(\zeta ,\tau )}{\partial \zeta }|}_{\zeta =0^{+}}=-1,$$

which confirms that the boundary condition (9) was satisfied. However, by approaching to the limit ζ→∞ in the solution (25), we have confirmed the fulfillment of the condition of the temperature fade (10). In addition, it was numerically established that the initial condition (11) was met. In particular, according to the Formula (25), on the heated surface *ζ* = 0 at the initial point in time, the dimensionless temperature rise is zero if the following occurs:

(32) $$\sum_{n=1}^{\infty }\frac{1}{\mu_{n}{}^{2}}\;=\frac{1}{4}.$$

Based on the calculations, it was found that the sum of the first 10^2^ components of the series on the left-hand side of Equation (32) is equal to 0.248985. 

In addition to the exact (25), the appropriate asymptotic solutions to the problem at small and large values of the Fourier number *τ* (dimensionless time) were also found.

*Small values*τ (*large values of the parameter* p). Taking into account that, in Formulas (17) and (18), the first components of the asymptotic of modified Bessel functions at large values of the argument [35] were as follows:

(33) $$I_{0}(x)≅\frac{e^{x}}{\sqrt{2\pi x}}\left(1+\frac{1}{8x}+\ldots \right),\;I_{1}(x)≅\frac{e^{x}}{\sqrt{2\pi x}}\left(1+\frac{3}{8x}+\ldots \right),$$

the transformed solution (16) was written as follows:

(34) $${\bar{\Theta }}^{*}(\zeta ,p)=e^{-\frac{1}{4}\gamma^{*}\zeta }\frac{e^{-b\sqrt{p}}}{p\sqrt{p}},\;b=\frac{2}{\gamma^{*}}\left(1-e^{-\frac{1}{2}\gamma^{*}\zeta }\right),\;\zeta \geq 0.$$

Using the relation [38] as follows:

(35) $$L^{-1}\left[\frac{e^{-b\sqrt{p}}}{p\sqrt{p}};\;\tau \right]=2\sqrt{\tau }\;\mathrm{ierfc}\;\left(\frac{b}{2\sqrt{\tau }}\right),$$

from the Formula (34), the following form of dimensionless temperature rise at the initial moments of heating was obtained:

(36) $$\Theta^{*}(\zeta ,\tau )≅2e^{-\frac{1}{4}\gamma^{*}\zeta }\sqrt{\tau }\;\mathrm{ierfc}\;\left(\frac{b}{2\sqrt{\tau }}\right),\;\zeta \geq 0,\;0\leq \tau <<1,$$

where $\mathrm{ierfc}(x)=\pi^{-\frac{1}{2}}e^{-x^{2}}-x\mathrm{erfc}(x)$, erfc(x)=1−erf(x), erf(x) is the Gauss error function [35].

At ζ=0 from the solution (36), a known result for the evolution of the temperature on the heated surface of a homogeneous semi-space was obtained [18] as follows:

(37) $$\Theta^{*}(\tau )≅2\sqrt{\frac{\tau }{\pi }},\;0\leq \tau <<1,$$

*Large values*τ*(small values of the parameter*p*).* By including the first two components in the distributions (22), from the transformed solution (16)–(18) it follows that:

(38) $${\bar{\Theta }}^{*}(\zeta ,p)≅\frac{e^{-\gamma^{*}\zeta }}{\gamma^{*}}\left[\frac{1}{p}-\left(1-\frac{1}{2}e^{-\gamma^{*}\zeta }\right)\frac{1}{\left(\gamma^{*}{}^{2}+p\right)}\right],\;\zeta \geq 0.$$

Considering the relations [38] as follows:

(39) $$L^{-1}[p^{-1};\;\tau ]=1,\;L^{-1}[{\left(\gamma^{*}{}^{2}+p\right)}^{-1};\;\tau ]=e^{-\gamma^{*}{}^{2}\tau },$$

from the solution (38), the following asymptotic representation for the dimensionless temperature rise at large values of time was obtained:

(40) $$\Theta^{*}(\zeta ,\tau )≅\frac{e^{-\gamma^{*}\zeta }}{\gamma^{*}}\left[1-\left(1-\frac{1}{2}e^{-\gamma^{*}\zeta }\right)e^{-\gamma^{*}\tau }\right],\;\zeta \geq 0,\;\tau >>1.$$

The temperature rise of the heated surface was found, substituting to the formula (40) ζ=0 as follows:

(41) $$\Theta^{*}(\tau )≅\frac{1}{\gamma^{*}}\left(1-\frac{1}{2}e^{-\gamma^{*}\tau }\right),\;\tau >>1.$$

## 3. Heating of the FGM Semi-Space by Heat Flux with the Intensity Linearly Decreasing in Time

As presented above, the exact (25), asymptotic (36), and (40) solutions were obtained at a constant intensity of heat flux of $q=q_{0}$. This section concerns the heating of the surface of the semi-space by heat flux with the following time profile of intensity:

(42) $$q(t)=q_{0}q^{*}(t),\;q^{*}(t)=1-\frac{t}{t_{s}},\;0\leq t\leq t_{s},$$

where $t_{s}$ is the stop moment of heating. The evolution of the heat flux intensity (42) corresponds to the temporal profile of the specific friction power during braking with constant deceleration [39]. The corresponding dimensionless temperature rise of ${\hat{\Theta }}^{*}$ was searched based on Duhamel’s theorem [40] as follows:

(43) $${\hat{\Theta }}^{*}(\zeta ,\tau )=\frac{\partial }{\partial \tau }\int_{0}^{\tau }q^{*}(\tau -s)\Theta^{*}(\zeta ,s)ds,\;\zeta \geq 0,0\leq \tau \leq \tau_{s},$$

where $\Theta^{*}$ is the dimensionless temperature rise (25)–(27), $q^{*}(\tau )$ is the function (42), and $\tau_{s}$ is the dimensionless stop time as follows:

(44) $$\tau_{s}=\frac{kt_{s}}{a^{2}}.$$

After the integration with the next differentiation on the right-hand side of Formula (42), the following was obtained:

(45) $${\hat{\Theta }}^{*}(\zeta ,\tau )=\frac{1}{\gamma^{*}}e^{-\frac{1}{2}\gamma^{*}\zeta }\left[q^{*}(\tau )\;e^{-\frac{1}{2}\gamma^{*}\zeta }-4\sum_{n=1}^{\infty }\frac{J_{1}(\mu_{n}e^{-\frac{1}{2}\gamma^{*}\zeta })}{\mu_{n}^{2}J_{1}(\mu_{n})}\;G_{n}(\tau )\right],\zeta \geq 0,0\leq \tau \leq \tau_{s},$$

where

(46) $$G_{n}(\tau )=e^{-\lambda_{n}\tau }-\frac{\left(1-e^{-\lambda_{n}\tau }\right)}{\lambda_{n}\tau_{s}},$$

the coefficients $\lambda_{n}$ were determined from Formula (26), and the numbers $\mu_{n}$, n=1,2,…, are the single positive roots of Equation (27).

By putting ζ=0 in the solution (45), the following formula was used to determine the evolution of the dimensionless temperature rise of the heated surface that was obtained:

(47) $${\hat{\Theta }}^{*}(\tau )=\frac{1}{\gamma^{*}}\left[q^{*}(\tau )-4\sum_{n=1}^{\infty }\frac{G_{n}(\tau )}{\mu_{n}^{2}}\;\right],\;0\leq \tau \leq \tau_{s}.$$

## 4. The Heat Partition Ratio

We will generalize the results that were obtained above to the case of two (l=1,2) FGM semi-spaces that exponentially increased with the distance from the surface z=0 of thermal conductivity as follows:

(48) $$K_{l}(z)=K_{l,0}e^{\gamma_{l}z},\;z\geq 0,\;K_{l,0}\equiv K_{l}(0),\;\gamma_{l}\geq 0,\;l=1,2.$$

**Figure 2:**
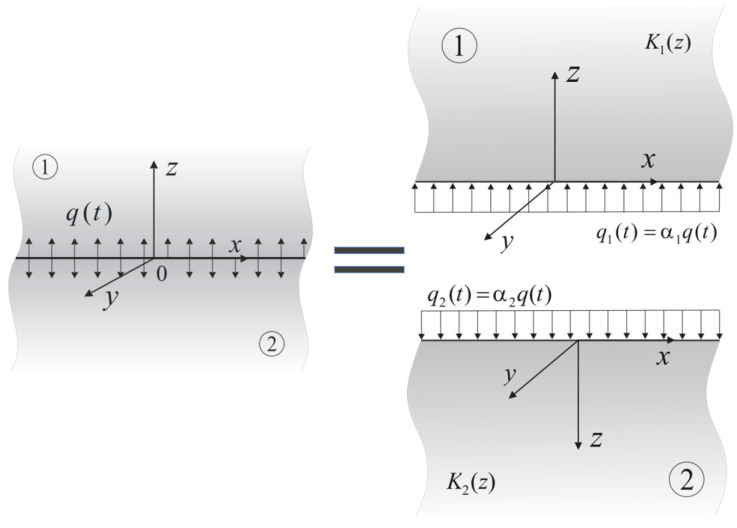
Scheme of separation of the friction pair elements.

The surfaces of z=0 of each semi-space were heated with the heat flux at the following intensities:

(49) $$q_{l}(t)=\alpha_{l}q(t),\;0\leq t\leq t_{s},\;l=1,2,\;\alpha_{1}=\alpha ,\;\alpha_{2}=1-\alpha ,$$

where q(t) is the function (42) and α is the unknown heat partition ratio (HPR) (Figure 2).

Taking into account the form of the solutions (45) and (46), the temperature field in each semi-space heated by heat fluxes of intensity (49) respectively, was written in the following form:

(50) $$T_{l}(z,t)=T_{0}+\Theta_{l,0}{\hat{\Theta }}_{l}^{*}(\zeta_{l},\tau^{*}),\;z\geq 0,\;0\leq t\leq t_{s},\;l=1,2,$$

where

(51) $${\hat{\Theta }}_{l}^{*}(\zeta_{l},\tau^{*})=\frac{1}{\gamma_{l}^{*}}e^{-\frac{1}{2}\gamma_{l}^{*}\zeta_{l}}\left[\left(1-\tau^{*})\;e^{-\frac{1}{2}\gamma_{l}^{*}\zeta_{l}}-4\sum_{n=1}^{\infty }\frac{J_{1}(\mu_{n}e^{-\frac{1}{2}\gamma_{l}^{*}\zeta_{l}})}{\mu_{n}^{2}J_{1}(\mu_{n})}\;G_{l,n}(\tau^{*}\right)\right],\;\zeta_{l}\geq 0,\;0\leq \tau^{*}\leq 1,$$

(52) $$G_{l,n}(t^{*})=e^{-\lambda_{l,n}\tau^{*}}-\frac{\left(1-e^{-\lambda_{l,n}\tau^{*}}\right)}{\lambda_{l,n}\tau_{l,s}},$$

(53) $$\lambda_{l,n}={\left(\frac{1}{2}\gamma_{l}^{*}\mu_{n}\right)}^{2}\tau_{l,s},$$

(54) $$\tau^{*}=\frac{t}{t_{s}},\;\zeta_{l}=\frac{z}{a_{l}},\;\tau_{l,s}=\frac{k_{l}t_{s}}{a_{l}^{2}},\;\gamma_{l}=\frac{\gamma_{l}^{*}}{a_{l}},\;k_{l}=\frac{K_{l,0}}{c_{l}\rho_{l}},\;{\hat{\Theta }}_{l}^{*}=\frac{{\hat{\Theta }}_{l}}{\Theta_{l,0}},\;\Theta_{l,0}=\frac{\alpha_{l}q_{0}a_{l}}{K_{l,0}},l=1,2,$$

$c_{l}$, $\rho_{l}$ are the specific heat capacity and the density of the materials, respectively, $\mu_{n}>0$, n=1,2,… are the single roots of Equation (27).

Substituting z=0 ($\zeta_{l}=0$) to the solution (51)–(54), the change in the temperature on the heated surface was obtained in the following form:

(55) $$T_{l}(t)=T_{0}+\Theta_{l,0}{\hat{\Theta }}_{l}^{*}(\tau^{*}),\;0\leq t\leq t_{s},\;l=1,2,$$

where

(56) $${\hat{\Theta }}_{l}^{*}(\tau^{*})=\frac{1}{\gamma_{l}^{*}}\left[1-\tau^{*}-4\sum_{n=1}^{\infty }\frac{G_{l,n}(\tau^{*})}{\mu_{n}^{2}}\right],\;0\leq \tau^{*}\leq 1.$$

The coefficients of $\Theta_{l,0}$ (54) in Formulas (50) and (55) contain an unknown heat partition ratio of α. In order to determine it, the equality condition of the mean temperature in time on the heated surfaces was used as follows:

(57) $${\tilde{T}}_{1}={\tilde{T}}_{2},$$

where

(58) $${\tilde{T}}_{l}=\frac{1}{t_{s}}\int_{0}^{t_{s}}T_{l}(t)dt,\;l=1,2,$$

Considering the temperature on the heated surfaces (55) and (56) in the Formula (58), after integration was found as follows:

(59) $${\tilde{T}}_{l}=T_{0}+\Theta_{l,0}{\tilde{\Theta }}_{l}^{*},\;l=1,2,$$

where

(60) $${\tilde{\Theta }}_{l}^{*}=\frac{1}{2\gamma_{l}^{*}}\left(1-8\sum_{n=1}^{\infty }\frac{{\tilde{G}}_{l,n}}{\mu_{n}^{2}}\right),\;$$

(61) $${\tilde{G}}_{l,n}=\frac{\left(1-e^{-\lambda_{l,n}}\right)}{\lambda_{l,n}}\left(1+\frac{1}{\lambda_{l,n}\tau_{l,s}}\right)-\frac{1}{\lambda_{l,n}\tau_{l,s}},\;l=1,2,$$

and the coefficients of $\lambda_{l,n}$ were determined from the formula (53).

Substituting the mean temperature (59)–(61) to Equation (57), the HPR was obtained in the following form:

(62) $$\alpha =\frac{K^{*}}{a^{*}{\tilde{\Theta }}^{*}+K^{*}},$$

where

(63) $$a^{*}=\frac{a_{1}}{a_{2}},\;K^{*}=\frac{K_{1,0}}{K_{2,0}},\;{\tilde{\Theta }}^{*}=\frac{{\tilde{\Theta }}_{1}^{*}}{{\tilde{\Theta }}_{2}^{*}}.$$

If the effective heating depths are determined from the Formula [9] as follows:

(64) $$a_{l}=\sqrt{3k_{l}t_{s}},\;l=1,2,$$

then from Formula (54) it follows that the dimensionless heating time of each semi-space is equal to $\tau_{l,s}=3^{-1}≅0.333$, and from Formulas (62) and (63) it follows that:

(65) $$\alpha =\frac{K_{\varepsilon }}{{\tilde{\Theta }}^{*}+K_{\varepsilon }},$$

where

(66) $$K_{\varepsilon }=\frac{K^{*}}{\sqrt{k^{*}}},\;k^{*}=\frac{k_{1}}{k_{2}}.$$

Omitting the component containing a series, on the right-hand side of Formula (60), i.e., assuming the following:

(67) $${\tilde{\Theta }}_{l}^{*}≅\frac{1}{2\gamma_{l}^{*}},\;l=1,2,$$

where

(68) $$\alpha ≅\frac{K^{*}\gamma^{*}}{a^{*}+K^{*}\gamma^{*}},$$

and Formula (65) is as follows:

(69) $$\alpha ≅\frac{K_{\varepsilon }\gamma^{*}}{1+K_{\varepsilon }\gamma^{*}},$$

where

(70) $$\gamma^{*}=\frac{\gamma_{1}^{*}}{\gamma_{2}^{*}}.$$

With the same effective heating depths ($a^{*}=1$) and dimensionless gradients of materials ($\gamma^{*}=1$) from Formula (68), the known Block’ result [20] as follows:

(71) $$\alpha ≅\frac{K^{*}}{1+K^{*}},$$

and from Formula (69), the classic Charron’s Formula [41] was achieved as follows:

(72) $$\alpha ≅\frac{K_{\varepsilon }}{1+K_{\varepsilon }},$$

which is often used in analytical and numerical modeling of the frictional heating of homogeneous materials during braking [42,43,44]. For the same materials of both semi-spaces ($K^{*}=k^{*}=\gamma^{*}=a^{*}=1$), all of the above obtained formulas give the value of the heat partition ratio equal to α=0.5.

It should be noted that the received formulas for the determination of HPR need to be verified in the future with appropriate experimental data. Due to the lack of such opportunity at that moment, the authors would be grateful for the provision of such data or for carrying out the cooperative research. This would also allow us to establish the limits of the applicability of the formulas. However, already at this stage of research, we can assume the possibility of the practical use of the obtained formulas to determine HPR, due to the usage of the classic methodology that has been approved for homogeneous materials. Furthermore, the experimentally confirmed formulas for homogeneous materials can be obtained from the proposed solution for FGMs, as a results of the proper limit approach.

An important element of the most general Formula (62) for determining the HPR are the dimensionless time-averaged temperature rises Formulas (60) and (61) found in the case of the heat flux intensity linearly decreasing in time. This case is often considered when calculating the temperature of the brake systems that are operating in the mode with a sudden increase in the contact pressure to the nominal value at the beginning of braking. A classification of the remaining heat flux intensity temporal profiles has been proposed for homogeneous materials, without and with consideration of the contact pressure rise [45,46]. Obtaining the appropriate solutions in the case of FGM is also one of the directions of our research in the future.

## 5. Example of Calculation of the Heat Partition Ratio for an FGM Couple

Calculations were performed for two semi-limited bodies made of two-component FGMs. The first (l=1) element forms aluminum oxide Al_2_O_3_ (base, m=0) and copper Cu (core, m=1), and the other (l=2) contains zircon dioxide ZrO_2_ (base, m=0) and titanium alloy Ti-6Al-4V (core, m=1). The thermo-physical properties of these materials are demonstrated in Table 1.

The other operating parameters are the nominal heat flux intensity of $q_{0}=3.78\;\;\mathrm{MW}\;m^{-2}$, the braking time of $t_{s}=121$ s, and the temperature at the initial time moment of $T_{0}=20\;{}^{\circ}C$ [32]. The dimensionless material gradient parameters of each element were calculated from the relation [47] as follows:

(73) $$\gamma_{l}^{*}=\ln\left(\frac{K_{l,1}}{K_{l,0}}\right),\;l=1,2.$$

Obtaining the values of $\gamma_{1}^{*}=2.38$, $\gamma_{2}^{*}=1.26$. The specific heat capacity and density of materials of the heated elements were determined according to the following mixture law:

(74) $$c_{l}=c_{l,1}v+(1-v)c_{l,0},\;\rho_{l}=\rho_{l,1}v+(1-v)\rho_{l,0},\;l=1,2,$$

where v is the volume fractions of the base and core components. For their equal participation (v=0.5), the following properties of FGM were found:

(75) $$c_{1}=437.32\;\;J\;{\mathrm{kg}}^{-1}K^{-1},\;\rho_{1}=6469.42\;\mathrm{kg}m^{-3},\;k_{1}=1.32\cdot 10^{-5}\;m^{2}s^{-1},$$

(76) $$c_{2}=495.46\;\;J\;{\mathrm{kg}}^{-1}K^{-1},\;\rho_{2}=5266.96\;\mathrm{kg}m^{-3},\;k_{2}=7.43\cdot 10^{-7}\;m^{2}s^{-1}.$$

From Equation (64) the effective depths of the heat penetration were determined as $a_{1}=21.8\;\mathrm{mm}$, $a_{2}=5.2\;\mathrm{mm}$. Next, based on Formulas (63), (66), and (70), the values of the dimensionless parameters were calculated as follows: $a^{*}=4.208$, $K^{*}=19.196$, $k^{*}=17.706$, $K_{\varepsilon }=4.562$, $\gamma^{*}=1.883$. This allowed for an estimation by means of Equations (60), (61) dimensionless, time-averaged temperature rises of ${\tilde{\Theta }}_{1}^{*}=0.329$, ${\tilde{\Theta }}_{2}^{*}=0.703$, and their ratio of ${\tilde{\Theta }}^{*}=0.468$ (63).

**Figure 3:**
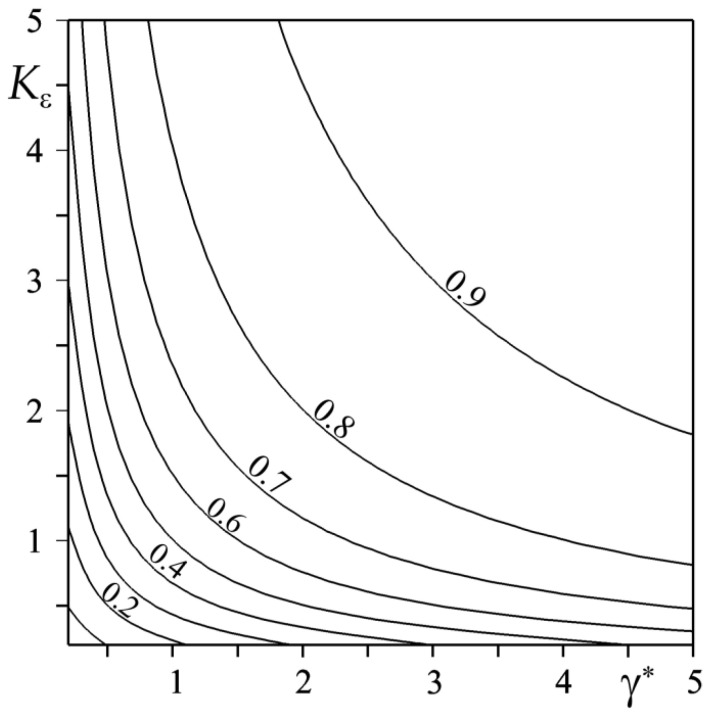
Isolines of the heat partition ratio α (69) in the coordinate system ($\gamma^{*}$, $K_{\varepsilon }$).

By substituting the found parameter values successively to the right-hand sides of Formulas (65) and (69), the proper values of the HPR were calculated as α=0.907 and α=0.896. A slight (1.2%) difference in the obtained results allowed us to analyze the influence of the dimensionless parameters of the thermal activity $K_{\varepsilon }$ (66) and the relative gradient of the FGMs $\gamma^{*}$ (70) on the HPR value (Figure 3), on the basis of Formula (69) only. For a fixed value of the thermal activity coefficient $K_{\varepsilon }$, a rise of the parameter $\gamma^{*}$ causes an increase in the amount of heat that is directed to the first element of the heated couple. Conversely, by increasing the thermal activity of the friction pair at a predetermined value $\gamma^{*}$, the amount of heat that is directed to the first element increases.

**Figure 4:**
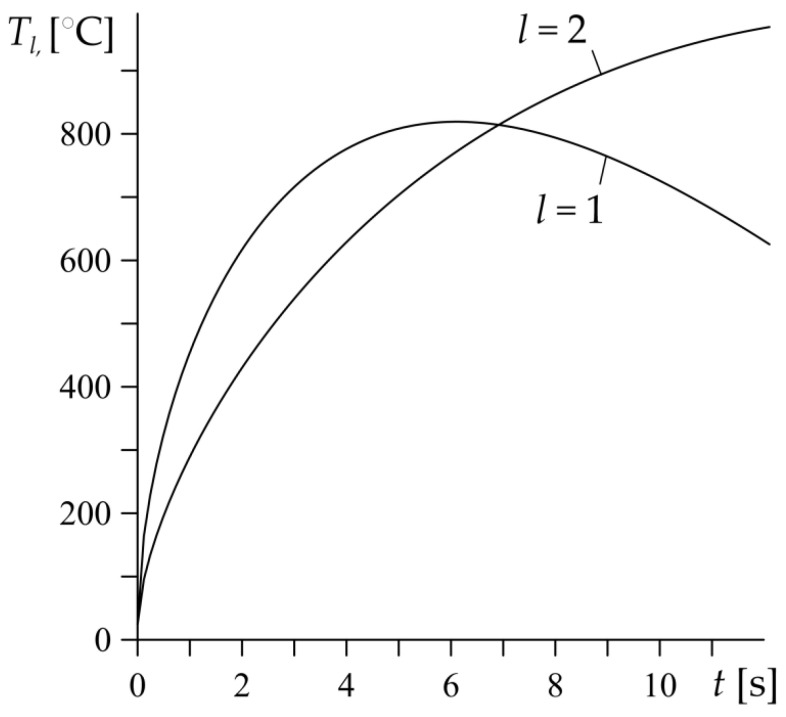
Evolutions of temperature $T_{l}$, l=1,2 (55) and (56) on the heated surfaces z=0 of the considered semi-spaces made of FGMs.

For the HPR values $\alpha_{1}=0.896$, $\alpha_{2}=0.104$ (49), from Equation (54), the scaling factors of the temperature rises were determined to be $\Theta_{1,0}=2034\;\;{}^{\circ}C$ and $\Theta_{2,0}=1080\;\;{}^{\circ}C$. Then, based on the relations Formulas (55) and (56) the evolutions of the temperature on the heated surfaces of each element were found (Figure 4).

It has been established that the temporal profiles of the temperature of the elements are different, in particular in the final stage of the heating process. The time course of the temperature of the first element (l=1, Al_2_O_3_–Cu) is typical for the evolution of the friction surface temperature during braking with a constant deceleration—a rapid increase in the temperature at the beginning of braking, reaching its maximum value in the middle of the process, followed by a temperature reduction until the standstill. However, in the second element (l=2, ZrO_2_–Ti–6Al–4V) a rise of temperature on the heated surface is monotonic during the whole process. Such temperature behavior is decisively influenced by the thermo-physical properties of the component materials of each element. In the functionally graded friction couple under consideration, the materials of both of the components of the first element have a significantly greater ability to dissipate the heat from the heated surface than the materials of the second element (Table 1). Moreover, this is confirmed, by the values of the coefficients $\alpha_{1}=0.896$, $\alpha_{2}=0.104$, which prove that the first element absorbs almost 90%, and the second only slightly more than 10% of the entire heat flux intensity q (42). Due to the low thermal conductivity of the component materials of the second element, in particular the zircon dioxide, the temperature of the heated surface of this element continues to rise during heating, even with a linearly decreasing intensity of the heat flux.

The results that are demonstrated in Figure 3 and Figure 4 were obtained by using the HPR value α=0.896 that was found from the dependency (69). The approximated formula for determining the α (69), as well as the exact Equation (65), was obtained by assuming that the effective depths of the heating of the elements $a_{l}$, l=1,2 were determined from the empirical Formula (64), so the corresponding values of the Fourier numbers $\tau_{l,s}$, l=1,2 (54) were the same and equal to 1/3. The introduction of Formula (64) to the model is justified when the thickness of the heated element is greater than the appropriate value *a_l_*, *l* = 1, 2 [2]. In general, for the determination of the temperature mode at the design stage of the brake, the Fourier numbers $\tau_{l,s},\;l=1,2$ are given, and the effective heating depths based on the relation (54) are calculated in the following form:

(77) $$a_{l}=\sqrt{\frac{k_{l}t_{s}}{\tau_{l,s}}},\;l=1,2.$$

**Figure 5:**
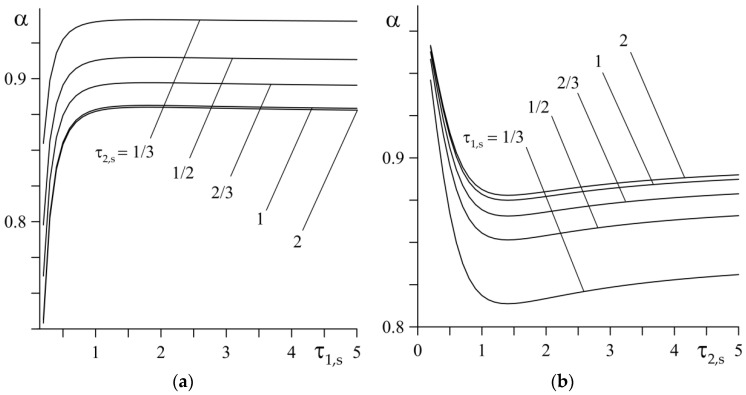
Dependences of the heat partition ratio α (62) on: (**a**) Fourier number $\tau_{1,s}$ for different values of $\tau_{2,s}$; (**b**) Fourier number $\tau_{2,s}$ for different values of $\tau_{1,s}$.

With consideration of Equation (75) for the selected friction couple and the established time of heating (braking) $t_{s}=12.1\;s$ the ratio of the depths of heating $a^{*}$ (63) is determined from the following relation:

(78) $$a^{*}=\sqrt{\frac{k^{*}}{\tau_{s}^{*}}},\;\tau_{s}^{*}=\frac{\tau_{1,s}}{\tau_{2,s}},$$

where $k^{*}$ is the ratio of the thermal diffusivities of the FGMs (66). By treating the Fourier numbers $\tau_{l,s}$, l=1,2 (54) as independent variables in Formula (62), their influence on the HPR α was investigated with the previously determined values of the dimensionless parameters, $K^{*}=19.196$, $k^{*}=17.706$, $\gamma_{1}^{*}=2.38$, and $\gamma_{2}^{*}=1.26$ for a given pair of elements. The dimensionless parameter ${\tilde{\Theta }}^{*}$ (63) included in Formula (62) was determined from Formulas (60) and (61), and the finding of $a^{{}_{*}}$ was made by using Formula (76). The results of the calculations are presented in Figure 5.

At a fixed value $\tau_{2,s}$, the HPR α quickly increases with increasing $\tau_{1,s}$, reaching its nominal value at $\tau_{1,s}≅1.5$ (Figure 5a). The largest nominal value α is achieved for $\tau_{1,s}=1/3$, i.e., when determining the effective heating depth using Formula (64). The results obtained at $\tau_{2,s}=1$ and $\tau_{2,s}=2$ practically coincide. A different nature of the HPR α change occurs with a fixed value of the Fourier number $\tau_{1,s}$ and increasing the values of $\tau_{2,s}$ (Figure 5b). By increasing $\tau_{2,s}$, the amount of heat that is absorbed by the first element decreases, reaching a minimum value at $\tau_{2,s}≅1.5$. It should be noted that the Fourier numbers $\tau_{l,s}$, l=1,2 (54) are dimensionless input parameters that play an important role in estimating the temperature mode of the brakes. The designers calculate their values after determining the geometric parameters $a_{l}$, l=1,2 (63). In the quality of these parameters, they choose the thickness of the friction elements, or, if they are greater than the effective depth of the heat penetration (64), then the latter. Then, having previously found the maps of those, as shown in Figure 5, the designer can easily estimate the HPR of a given braking system.

## 6. Conclusions

A methodology was proposed in order to determine the heat partition ratio (HPR) in the braking systems with friction pair elements that were made of functionally graded materials (FGMs). The basis of this methodology is an exact solution to the initial-boundary problem of heat conduction that is formulated for the FGM semi-space that is heated on its surface by a heat flux with an intensity that decreases linearly with time and includes an unknown a priori HPR. For its determination, the condition of the equality of the time-averaged temperature on the heated surfaces of the two different semi-spaces was used.

As an example, the heat partition ratio was found for the FGM friction couple that consisted of Al_2_O_3_–Cu and ZrO_2_–Ti–6Al–4V. It was established that element Al_2_O_3_–Cu absorbs most of the heat that is generated due to friction (almost 90%). The maximum temperature on the friction surface of the Al_2_O_3_–Cu element is about 820 °C and is achieved in the middle of the braking time. However, the highest temperature of the friction surface of the ZrO_2_–Ti–6Al–4V element is achieved at the stop moment and amounts to 970 °C. Thus, the crucial influence on the evolution of the temperature in the functionally graded friction couple have the thermo-physical properties of the component materials.

Then, the simulation of the HPR dependency on the dimensionless parameters, such as the thermal activity of the friction couple, the gradient materials, and the Fourier numbers was carried out. It was found that increasing the values of the friction couple thermal activity or the gradient materials ratio causes an increase in the HPR. The scheme of the map development for a given functionally graded friction pair was proposed, which allows for a quick estimation of the HPR, depending on such input parameters as their Fourier numbers. The importance of having such maps for the designer is indicated by the results that have been obtained in the case of the homogeneous materials [9,28].

## Figures and Tables

**Table 1:** Thermo-physical properties of FGM components [32].

Element Subscript	Material Subscript, *m*	Material	Thermal Conductivity $K_{l,m}^{\left(0\right)},\;{\mathrm{Wm}}^{-1}K^{-1}$	Specific Heat Capacity $c_{l,m}^{\left(0\right)},\;J\;{\mathrm{kg}}^{-1}K^{-1}$	$\mathrm{Density}\;\rho_{l,m}^{\left(0\right)},\;{\mathrm{kgm}}^{-3}$
l=1	*base*, 0	Al_2_O_3_	37.24	727.29	3990.92
	*core*, 1	Cu	402.65	147.35	8947.92
l=2	*base*, 0	ZrO_2_	1.94	452.83	6102.16
	*core*, 1	Ti-6Al-4V	6.87	538.08	4431.79

## Data Availability

No new data were created or analyzed in this study. Data sharing is not applicable to this article.

## Nomenclature

a	Effective depth of heat penetration (m )
c	Specific heat capacity ($J\;{\mathrm{kg}}^{-1}K^{-1}$)
$f_{0}$	Coefficient of friction
$I_{k}(\cdot )$	Modified Bessel functions of the first kind of the *k*th order
$J_{k}(\cdot )$	Bessel functions of the first kind of the *k*th order
k	Thermal diffusivity ($m^{2}s^{-1}$)
K	Thermal conductivity ($Wm^{-1}K^{-1}$)
$K_{\varepsilon }$	Dimensionless coefficient of thermal activity of friction couple
p	Dimensionless parameter of the Laplace integral transform
q	Intensity of heat flux ($Wm^{-2}$)
$q_{0}$	Nominal intensity of the heat flux ($Wm^{-2}$)
t	Time (s)
$t_{s}$	Braking time (s)
T	Temperature (${}^{\circ }C$)
$T_{0}$	Initial temperature (${}^{\circ }C$)
v	Volume fraction of the material phases (dimensionless)
z	Spatial coordinate in axial direction (m )
α	Heat partition ratio
γ	Parameter of material gradient ($m^{-1}$)
$\gamma^{*}$	FGMs gradient ratio
Θ	Temperature rise (${}^{\circ }C$)
$\Theta^{*}$	Dimensionless temperature rise
$\Theta_{0}$	Scaling factor of temperature rise (${}^{\circ }C$)
ρ	Density ($\mathrm{kg}m^{-3}$)
$\tau^{*}$	Dimensionless time
$\tau_{s}$	Dimensionless braking time
ζ	Dimensionless spatial coordinate in axial direction
